# PairMotif: A New Pattern-Driven Algorithm for Planted (*l*, *d*) DNA Motif Search

**DOI:** 10.1371/journal.pone.0048442

**Published:** 2012-10-31

**Authors:** Qiang Yu, Hongwei Huo, Yipu Zhang, Hongzhi Guo

**Affiliations:** School of Computer Science and Technology, Xidian University, Xi’an, Shaanxi, China; Universitat Rovira i Virgili, Spain

## Abstract

Motif search is a fundamental problem in bioinformatics with an important application in locating transcription factor binding sites (TFBSs) in DNA sequences. The exact algorithms can report all (*l*, *d*) motifs and find the best one under a specific objective function. However, it is still a challenging task to identify weak motifs, since either a large amount of memory or execution time is required by current exact algorithms. A new exact algorithm, PairMotif, is proposed for planted (*l*, *d*) motif search (PMS) in this paper. To effectively reduce both candidate motifs and scanned *l*-mers, multiple pairs of *l*-mers with relatively large distances are selected from input sequences to restrict the search space. Comparisons with several recently proposed algorithms show that PairMotif requires less storage space and runs faster on most PMS instances. Particularly, among the algorithms compared, only PairMotif can solve the weak instance (27, 9) within 10 hours. Moreover, the performance of PairMotif is stable over the sequence length, which allows it to identify motifs in longer sequences. For the real biological data, experimental results demonstrate the validity of the proposed algorithm.

## Introduction

Motif search plays an important role in gene finding and gene regulation relationship understanding. Taking a survey of recent developments in motif recognition algorithms, Das and Dai [1] pointed out that DNA motif search would still be an opening challenge for researchers. In this paper, we focus on the planted (*l*, *d*) motif search [2], a widely used model for motif finding:

### Planted (*l*, *d*) Motif Search (PMS)

Given a set of *n*-length sequences *S* = {*s*_1_, *s*_2_, …, *s_t_*} over the alphabet {A, T, C, G} and nonnegative integers *l* and *d*, satisfying 0≤ *d* < *l* < *n*, the task is to find an *l*-mer (i.e., an *l*-length string) *m*, called a motif, such that each sequence *s_i_* contains an *l*-mer *m_i_* differing from *m* in at most *d* positions.

In the PMS problem, typical values of *t* and *n* are 20 and 600; then, various combinations of *l* and *d* correspond to different instances of PMS. The instances where the value of *d* is large in relation to the value of *l* are called weak instances, which are difficult to be solved. For example, the instances (15, 4) and (18, 6) are well-known weak instances [3].

Numerous recognition algorithms, either approximate or exact, have been proposed. The initialization of most approximate algorithms is selecting start sites randomly to begin iterations, which makes them easily fall into a local optimum. Gibbs Sampling [4] and MEME [5] are classic algorithms in this approach, and they usually use several groups of start sites as the initial states to avoid the local optimum. PROJECTION [6] partitions all *l*-mers in *S* into many buckets, and selects some valid buckets that contain several occurrences of the desired motif and little else. Then EM algorithm is used to refine the valid buckets. MotifCut [7] is a graph-theoretic approach in which motif finding is formulated as a search for the maximum density subgraph. MCEMDA [8], a Monte Carlo version of the EM motif finding algorithm, starts from an initial model, and then it iteratively performs Monte Carlo simulation and parameter update until convergence. SBaSeTraM [9] is a Bayesian search method obtained by combining two models, namely a foreground model and a background model, which describe the distribution of sequences under the alternative hypothesis that there is a TFBS and under the null hypothesis that there is no TFBS at the site, respectively. Vine [10], the recent method, is a polynomial-time heuristic algorithm for motif search based on WINNOWER [2]. Generally, the approximate algorithms are able to produce results in a short time, but they cannot guarantee global optimality.

Exact recognition algorithms are guaranteed to obtain the best solution, although exponential running time is required in the worst case due to the NP-hard nature of PMS [11]. It is therefore meaningful to design the exact algorithm with a small time overhead. According to the search space of PMS, there are two types of exact recognition algorithms. One is the exact algorithms based on alignment matrix, which test all (*n* − *l* +1)*^t^* possible combinations of motif positions in each of sequences to find the one that yields the highest score. The other is the exact algorithms based on pattern, which verify all 4*^l^* possible patterns to find the one that appears in all *t* sequences with the minimum number of mutations. Although the two types of exact algorithms produce the consistent results [12], the initial search space of the latter is much smaller than that of the former. Therefore, the research mainly concentrates on the latter in recent years, and the associated algorithms [13]–[22] are called pattern-driven algorithms.

All pattern-driven algorithms aim to reduce candidate motifs through various means. However, no single algorithm is superior to others on all PMS instances. Being the fastest in the family of suffix tree algorithms [13]–[18], RISOTTO [18] shows competitive execution time, but its performance drops significantly with the increase of the motif length. PMSP [19] adopts the following idea: for each *l-*mer *x* in *s*_1_, it generates *d*-neighbors of *x*, and then verifies if each *d*-neighbor *y* is a motif by checking whether there are *l-*mers in *s_i_* (2≤ *i* ≤ *t*) that are at distance *d* from *y*. PMSprune [20] is an improvement over PMSP obtained by using branch and bound, capable of solving many PMS instances in a short time. The iTriplet algorithm [21], which constructs multiple triplets of *l*-mers to reduce candidate motifs, is suitable for identifying long motifs (>20 nucleotides), but suffers from substantial computational requirements. PMS5 [22], whose main idea is to use integer programming to compute the common *d*-neighbors of three *l-*mers, is an efficient algorithm for solving weak PMS instances with the value of *l* about 20. But PMS5 is difficult to solve weak instances with large values of *l*, because of the substantial memory required for storing the results of all possible integer linear programs. There are some other methods for the exact algorithms, see for example: [23]–[25] etc.

Besides the search method, objective functions also play an important role in motif search. The analysis of wild-type and mutant Zif268 zinc fingers genes made by Bulyk et al. [26] indicated that there exists interdependence among positions in transcription factor binding sites for real biological data. If the objective function cannot express the interdependence, the obtained optimal solution may not possess a real biological significance. Li and Tompa [27] assessed and classified the objective functions used by existing tools, and they pointed out that an ideal objective function should assign the optimal score to the true motif. For the exact algorithms, the objective function is used to rank reported (*l*, *d*) motifs, and the prediction is done by selecting the motif with best score.

To effectively solve the PMS problem, a new pattern-driven algorithm, PairMotif, is proposed in this paper. First, motivated by the observation that the number of candidate motifs shared by two *l-*mers is dramatically affected by the distance between the two *l*-mers, multiple pairs of *l*-mers are carefully selected from input sequences to limit the total candidate volume. Second, a new filtering rule based on a pair of *l*-mers is designed to filter out more *l*-mers to be scanned in candidate verification. Third, a deterministic and efficient method for traversing candidate motifs is given to perform candidate verification. The experimental results demonstrate the efficiency and effectiveness of the proposed algorithm.

## Methods

### Ethics Statement

N/A. The ethics approval is not necessary for our study. That is because: 1) our study doesn’t make use of any human or vertebrate animal subjects and tissue; 2) our study focuses on faster algorithms for planted (*l*, *d*) motif search, which is a widely used computing model for DNA motif search, and our experiments are completed only by using computers.

### Notations and Definitions

The Hamming distance between two *l*-mers *x* and *x*′ is denoted by *d_H_*(*x*, *x*′). In PairMotif, all pairs of *l*-mers *x* and *x*′ that satisfy *d_H_*(*x*, *x*′) >2*d* are ignored, since the Hamming distance between two instances of the same motif must be less than or equal to 2*d* and 2*d* is less than *l*. Given an *l*-mer *x* and a sequence *s*, let *C*(*x*, *s*) be the set of all such *l*-mers *y* in *s* that *d_H_*(*x*, *y*) ≤2*d*.

**Figure 1:**
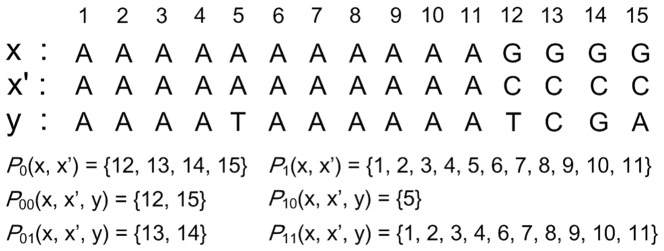
An example for partitioning positions in the alignment of two/three *l*-mers. This figure shows an example for partitioning positions in the alignment of two/three 15-mers.

Given two *l*-mers *x* and *x*′, let *P*_1_(*x*, *x*′) be the set of positions at which the corresponding characters are identical, and *P*_0_(*x*, *x*′) be the set of positions at which the corresponding characters are different. Given another *l*-mer *y*, the *l* positions in the alignment of *x*, *x*′ and *y* can be divided into four categories: *P*_00_(*x*, *x*′, *y*), *P*_01_(*x*, *x*′, *y*), *P*_10_(*x*, *x*′, *y*) and *P*_11_(*x*, *x*′, *y*). More precisely, for each position *i*, assume that it belongs to *P_ab_*(*x*, *x*′, *y*). Then, *a* is 1 if *x*[*i*] = *x*′[*i*], 0 otherwise; *b* is 1 if either *y*[*i*] = *x*[*i*] or *y*[*i*] = *x*′[*i*], 0 otherwise. Figure 1 shows an example for partitioning the positions in the alignment of two *l*-mers and that of three *l*-mers.

#### Definition 1

Given two *l*-mers *x* and *x*′, the candidate motifs shared by *x* and *x*′, *M_d_*(*x*, *x*′), is defined to be {*y*: |*y*| = *l*, *d_H_*(*y*, *x*) ≤ *d* and *d_H_*(*y*, *x*′) ≤ *d*}.

#### Definition 2

Given three *l*-mers *x*, *x*′ and *y* with *y* ∈ *M_d_*(*x*, *x*′), the mapping relation from *x* and *x*′ to *y*, *R*(*x*, *x*′, *y*), is defined to be a 2-tuple <*α*, *β*> = <|*P*_10_(*x*, *x*′, *y*)|, |*P*_00_(*x*, *x*′, *y*)|>. Furthermore, the mapping relation from *x* and *x*′ to *M_d_*(*x*, *x*′), *R*(*x*, *x*′), is defined to be 
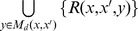
.

### PairMotif Algorithm

**Figure 2:**
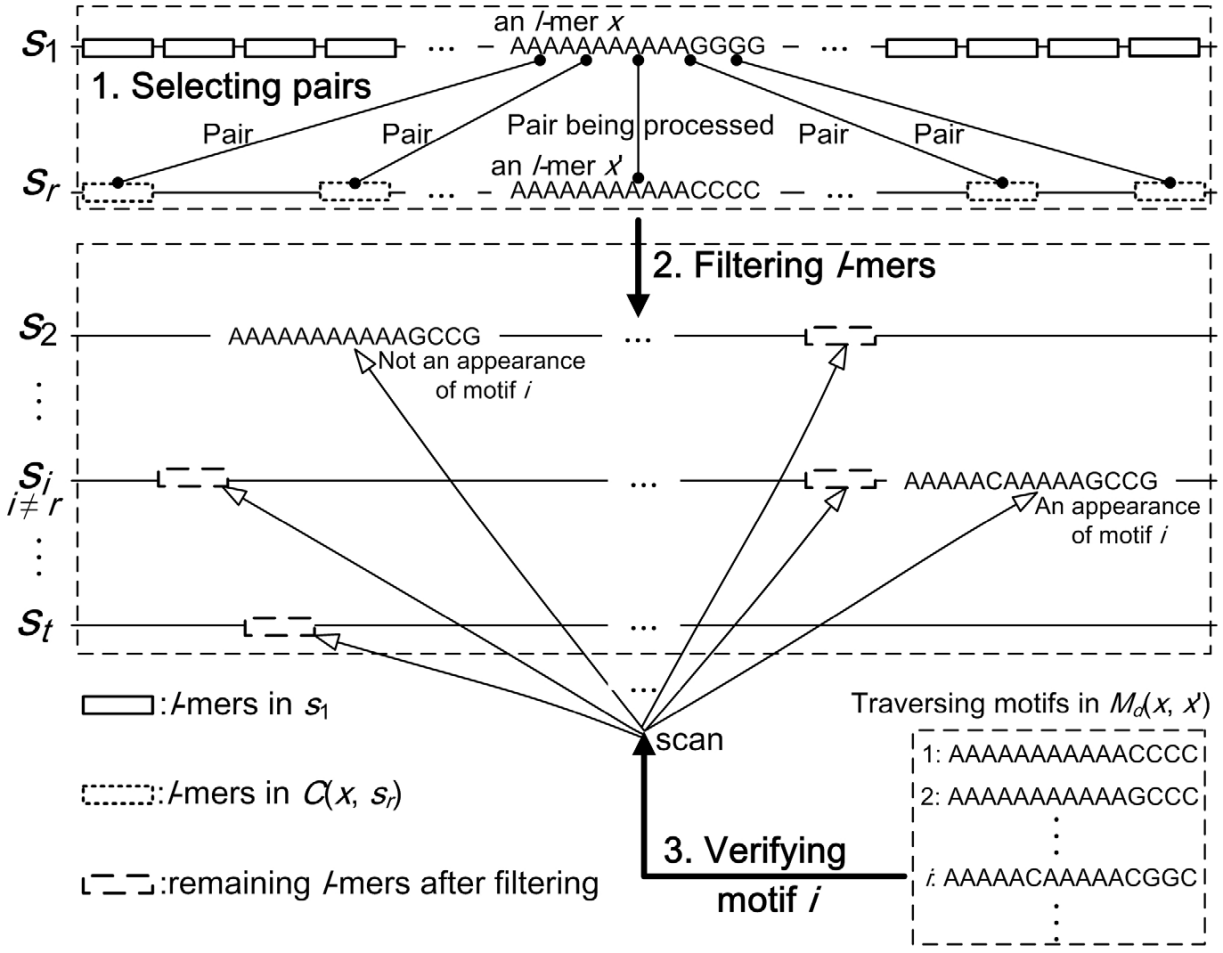
Illustration of the PairMotif algorithm. This figure takes the instance (15, 4) as an example to explain the process of PairMotif, which consists of three stages: selecting pairs, filtering *l*-mers and verifying candidate motifs.

As shown in Figure 2, PairMotif consists of the following three stages:

1. Selecting pairs. For each *l*-mer *x* in *s*_1_, select a reference sequence *s_r_* from *S* − {*s*_1_}. Then, multiple pairs of *l*-mers are formed by pairing the *l*-mer *x* and each *l*-mer *x*′ in *C*(*x*, *s_r_*). Note that, *s_r_* is selected with the most restrictive limit on the total number of candidate motifs in comparison with other sequences.
2. Filtering *l*-mers. For each selected pair of *l*-mers *x* and *x*′, use two filtering rules to filter out *l*-mers in *C*(*x*, *s_i_*) (2≤ *i* ≤ *t*, *i* ≠ *r*) that cannot be instances of any candidate motif in *M_d_*(*x*, *x*′). Let *C*′(*x*, *s_i_*) denote the set of the remaining *l*-mers in *C*(*x*, *s_i_*) after filtering.
3. Verifying candidate motifs. For each selected pair of *l*-mers *x* and *x*′, traverse each candidate motif *y* in *M_d_*(*x*, *x*′), and verify if *y* is a motif by checking whether there is an instance of *y* in each *C*′(*x*, *s_i_*).

Based on these three stages, the PairMotif algorithm is presented as follows.

### Algorithm PairMotif

**Input:** *l*, *d*, *S* = {*s*_1_, *s*_2_, …, *s_t_*}

**Output:** the (*l*, *d*) motif set *M*

1: *M* ← *Ф*

2: **for** each *l*-mer *x* in *s*_1_ **do**

3: Compute *C*(*x, s_i_*) (2≤ *i* ≤ *t*)

4: Select a reference sequence *s_r_* from *S* − {*s*_1_}

5: **for** each *x*′ ∈ *C*(*x, s_r_*) **do**

6: Form a pair of *l*-mers *x* and *x*′

7: Filter *l*-mers in *C*(*x, s_i_*) (2≤ *i* ≤ *t*, *i* ≠ *r*) and store the remaining *l*-mers in *C*′(*x, s_i_*)

8: **for** each *y* ∈ *M_d_*(*x, x*′) **do**

9: **if** for every *i* (2*≤ i ≤ t*, *i* ≠ *r*), there is a *y_i_* ∈ *C*′(*x, s_i_*) such that *d_H_*(*y, y_i_*) ≤ *d* **then**

10: Add *y* to *M*

11: Output *M*

In line 1, the set of (*l*, *d*) motifs *M* is initialized to an empty set. Lines 2–6 correspond to the stage of selecting pairs, in which *C*(*x, s_i_*) is obtained by calculating the Hamming distance from the *l*-mer *x* to all *l-*mers in *s_i_* (2≤ *i* ≤ *t*). Line 7 and lines 8–10 show the stages of filtering *l*-mers and verifying candidate motifs, respectively. PairMotif guarantees to discover all (*l*, *d*) motifs and outputs them in line 11.

Next, we explain key techniques for each stage.

### Stage 1: Selecting Pairs

For each *l-*mer *x* in *s*_1_, a reference sequence *s_r_* is required to form multiple pairs of *l*-mers. Specifically, *s_r_* is selected from *S* - {*s*_1_} by satisfying.
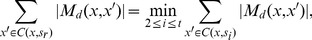
(1)where 
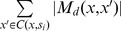
 denotes the number of candidate motifs determined by *x* and *s_i_*. After describing details of Stage 3, we will give the formula for calculating *|M_d_*(*x*, *x*′)|, which depends only on *d_H_*(*x*, *x*′) and parameters *l* and *d*. In experiments, the values of *|M_d_*(*x*, *x*′)| under different Hamming distances are cached to speed up pairs selection.

This selection method is valuable for limiting the total number of candidate motifs.

#### Observation 1

*|M_d_*(*x*, *x*′)| grows dramatically with the decrease of *d_H_*(*x*, *x*′).

Table 1 takes the instance (15, 4) as an example to show the values of *|M_d_*(*x*, *x*′)| under different *d_H_*(*x*, *x*′). For any four 15-mers *x*_1_, *x*_1_′, *x*_2_ and *x*_2_′, even though the difference between *d_H_*(*x*_1_, *x*_1_′) and *d_H_*(*x*_2_, *x*_2_′) is not large, *|M_d_*(*x*_1_, *x*_1_′)| and *|M_d_*(*x*_2_, *x*_2_′)| can differ by several times or more. For example, when *d_H_*(*x*_1_, *x*_1_′) = 8 and *d_H_*(*x*_2_, *x*_2_′) = 4, their difference is 4, whereas *|M_d_*(*x*_2_, *x*_2_′)| is about 94 times *|M_d_*(*x*_1_, *x*_1_′)|.

**Table 1:** *R*(*x*, *x*′) and |*M_d_*(*x*, *x*′)| for the instance (15, 4).

d_H_(x, x′)	R(x, x′)	|M_d_(x, x′)|
9–15	Ф	0
8	{<0,0>}	70
7	{<0,0>, <0,1>}	350
6	{<0,0>, <0,1>, <0,2>, <1,0>}	1190
5	{<0,0>, <0,1>, <0,2>, <0,3>, <1,0>, <1,1>}	2970
4	{<0,0>, <0,1>, <0,2>, <0,3>, <0,4>, <1,0>, <1,1>, <1,2>, <2,0>}	6600
3	{<0,0>, <0,1>, <0,2>, <0,3>, <1,0>, <1,1>, <1,2>, <1,3>, <2,0>, <2,1>}	13504
2	{<0,0>, <0,1>, <0,2>, <1,0>, <1,1>, <1,2>, <2,0>, <2,1>, <2,2>, <3,0>}	27316
1	{<0,0>, <0,1>, <1,0>, <1,1>, <2,0>, <2,1>, <3,0>, <3,1>}	42760
0	{<0,0>, <1,0>, <2,0>, <3,0>, <4,0>}	100636

This table shows the values of *R*(*x*, *x*′) and |*M_d_*(*x*, *x*′)| for the instance (15, 4) under different Hamming distances.

Based on this observation, given an *l*-mer *x* in *s*_1_, we can analyze how the number of candidate motifs changes with different *s_i_* in *S* - {*s*_1_}. On the one hand, there are a relatively small number of candidate motifs, if all *l*-mers in *s_i_* are at a relatively large Hamming distance from *x*. On the other hand, *x* and *s_i_* will lead to a huge number of candidate motifs, once there are several *l*-mers in *s_i_* at a very small Hamming distance from *x*. Our selection method limits the total candidate volume by preventing the occurrence of the latter case.

### Stage 2: Filtering l-mers

In PairMotif, for each selected pair of *l*-mers *x* and *x*′, two filtering rules below are used to determine if each *l*-mer *z* in *S* - {*s*_1_, *s_r_*} is a possible instance of a certain candidate motif in *M_d_*(*x*, *x*′):

#### Rule 1

If either *d_H_*(*z*, *x*) >2*d* or *d_H_*(*z*, *x*′) >2*d*, then *z* is not an instance of any candidate motif in *M_d_*(*x*, *x*′).

#### Rule 2

If there is no such a 2-tuple <*α*, *β*> ∈ *R*(*x*, *x*′) that satisfies *abs*(|*P*_10_(*x*, *x*′, *z*)|−*α*) + *abs*(|*P*_00_(*x*, *x*′, *z*)|−*β*) ≤ *d*, then *z* is not an instance of any candidate motif in *M_d_*(*x*, *x*′). The symbol *abs*(·) denotes the operation of taking the absolute value.

Rule 1 employs the widely used criterion that two instances of the same motif must not differ by more than 2*d* differences. Rule 2 realizes filtration from a different perspective (the proof of its correctness is included in the Text S1): it compares the distance with *d* in the case that there is an error value <*α*, *β*>. Therefore, Rule 2 can filter out some *l*-mers that cannot be filtered out by Rule 1. Let us take the instance (15, 4) as an example. Assume that there are three *l*-mers *x* (AAAAAAAGGGGGGGG), *x*′ (AAAAAAACCCCCCCC) and *z* (AAAAAAATTTTTTTT). By Rule 2, *z* can be filtered out successfully since *R*(*x*, *x*′) = {<0, 0>} and *abs*(|*P*_10_(*x*, *x*′, *z*)|−0) + *abs*(|*P*_00_(*x*, *x*′, *z*)|−0) = 0+8 = 8> *d*. But Rule 1 is invalid since *d_H_*(*x*, *x*′) = *d_H_*(*x*, *z*) = *d_H_*(*x*′, *z*) = 8≤2*d*. However, there also exist some *l*-mers that can be filtered out by Rule 1, but cannot be filtered out by Rule 2. For example, keep *x* and *x*′ unchanged, and let *z = *TTTTAAAGGGGGGGG. By Rule 1, *d_H_*(*x*′, *z*) = 12>2*d*, so *z* is filtered out. But by Rule 2, *z* cannot be filtered out since *abs*(|*P*_10_(*x*, *x*′, *z*)|−0) + *abs*(|*P*_00_(*x*, *x*′, *z*)|−0) = 4+0 = 4≤ *d*. Taking these considerations into account, we use these two rules to simultaneously perform filtration.

Moreover, the fact that most pairs of *l*-mers selected in Stage 1 have relatively large Hamming distances is conducive to filtering out more *l*-mers. This can be understood through Observation 1. The larger the value of *d_H_*(*x*, *x*′), the smaller the value of *|M_d_*(*x*, *x*′)|. Accordingly, given a random *l*-mer *z*, the probability that *z* is one of the *d*-neighbors of *M_d_*(*x*, *x*′) is relatively small when *d_H_*(*x*, *x*′) is relatively large.

On the basis above, most *l*-mers, which need to be compared with each candidate motif in Stage 3, are filtered out in advance.

### Stage 3: Verifying Motifs

For each selected pair of *l*-mers *x* and *x*′, candidate motifs in *M_d_*(*x*, *x*′) need to be traversed to perform candidate verification. This section gives a deterministic and efficient traversing method, rather than enumerating all possible *l*-mers.

At first, we discuss how to compute *R*(*x*, *x*′) given a pair of *l*-mers *x* and *x*′; *R*(*x*, *x*′) implies the approach to traversing candidate motifs in *M_d_*(*x*, *x*′). Assume that *y* is a candidate motif in *M_d_*(*x*, *x*′) with *R*(*x*, *x*′, *y*) = <*α*, *β*>. By Definition 2, we have *α* ≤ *l*−*d_H_*(*x*, *x*′), *β* ≤ *d_H_*(*x*, *x*′) and *d_H_*(*y*, *x*) + *d_H_*(*y*, *x*′) = 2*α* +2*β* + (*d_H_*(*x*, *x*′)−*β*). Furthermore, we have *d_H_*(*y*, *x*) + *d_H_*(*y*, *x*′) ≤2*d* because both *x* and *x*′ are instances of *y*. Thus, we obtain:
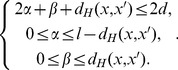
(2)


Obviously, the value of *R*(*x*, *x*′) is determined by *d_H_*(*x*, *x*′). Given the value of *d_H_*(*x*, *x*′), it is easy to compute *R*(*x*, *x*′) for the specified (*l*, *d*) instance by listing all 2-tuples <*α*, *β*> satisfying (2). Table 1 shows *R*(*x*, *x*′) for the instance (15, 4) under different Hamming distances.

**Figure 3:**
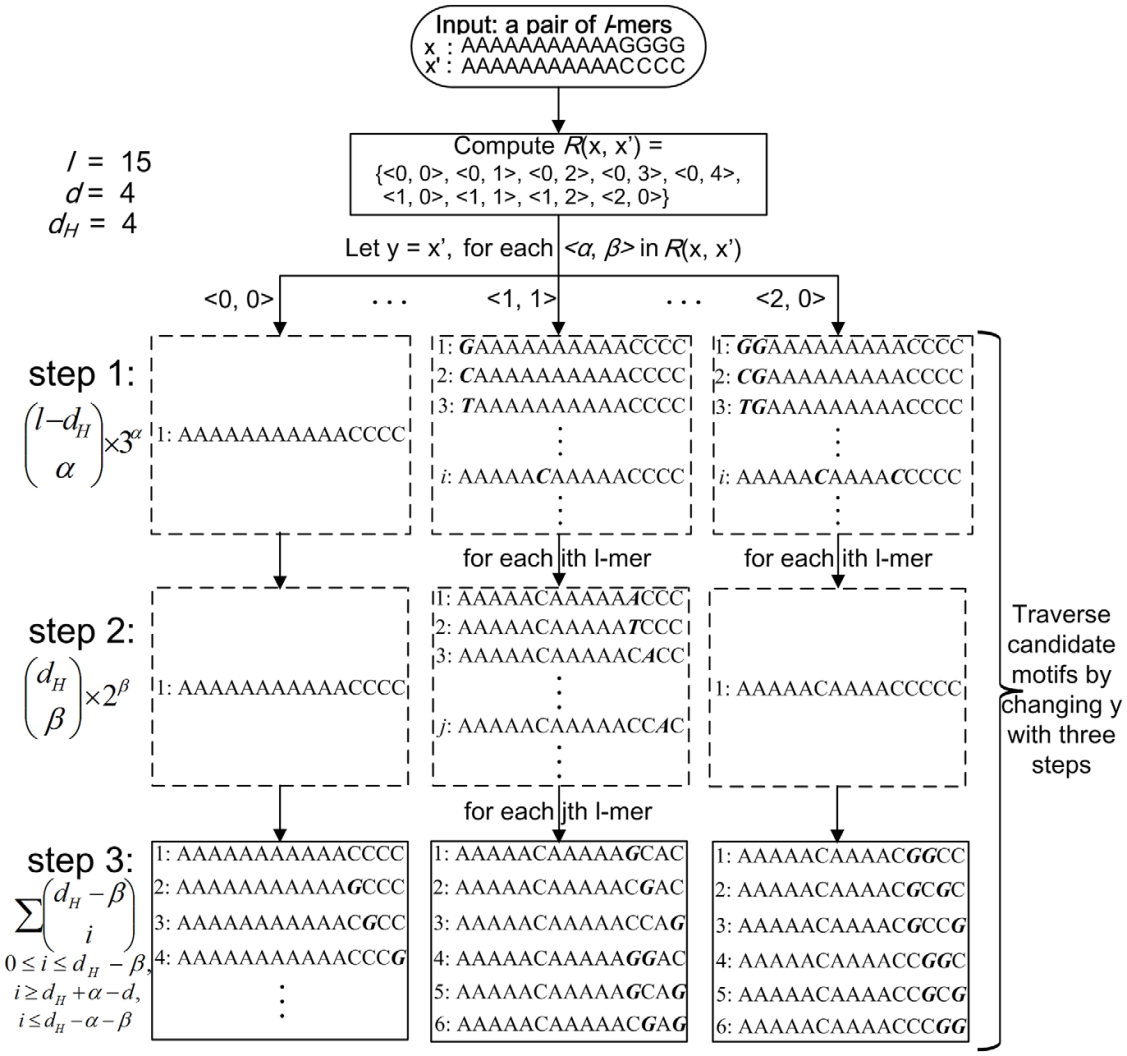
An example for traversing candidate motifs in *M_d_*(*x*, *x*′). This figure shows an example for traversing candidate motifs shared by two *l*-mers *x* and *x*′. After calculating *R*(*x*, *x*′), for each <*α*, *β*> in *R*(*x*, *x*′), let *y* = *x*′, and the process of traversing is implemented by changing *y* with three steps. First, select *α* positions from the positions where *x*[*i*] = *x*′ [*i*], and for each *i* of these *α* positions, change *y*[*i*] to one of the three characters different from *x*[*i*]. Second, select *β* positions from the positions where *x*[*i*] ≠*x*′ [*i*], and for each *i* of these *β* positions, change *y*[*i*] to one of the two characters different from *x*[*i*] and *x*′ [*i*]. Third, select a part of positions from the positions where *x*[*i*] ≠*x*′ [*i*] except for those selected in step 2, and change *y*[*i*] to *x*[*i*] for each *i* of these positions. The bold italic characters denote the changed positions in *y*.

Based on different 2-tuples in *R*(*x*, *x*′), candidate motifs in *M_d_*(*x*, *x*′) can be traversed in a simple way. *M_d_*(*x*, *x*′) consists of several mutually disjoint subsets with each subset sharing a different 2-tuple in *R*(*x*, *x*′); these subsets are visited one by one. As shown in Figure 3, for each <*α*, *β*> in *R*(*x*, *x*′), let *y* = *x*′, and then we traverse candidate motifs in the associated subset of *M_d_*(*x*, *x*′) by changing *y* with the following three steps. First, select *α* positions from *P*_1_(*x*, *x*′), and for each *i* of these *α* positions, change *y*[*i*] to one of the three characters different from *x*[*i*]; second, select *β* positions from *P*_0_(*x*, *x*′), and for each *i* of these *β* positions, change *y*[*i*] to one of the two characters different from *x*[*i*] and *x*′ [*i*]; third, select a part of positions from *P*_0_(*x*, *x*′) except for those selected in step 2, and change *y*[*i*] to *x*[*i*] for each *i* of these positions. Note that, in step 3, the number of selected positions should ensure *d_H_*(*x*, *y*) ≤ *d* and *d_H_*(*x*′, *y*) ≤ *d*.

According to the process of traversing candidate motifs, the size of *M_d_*(*x*, *x*′) is calculated as follows:
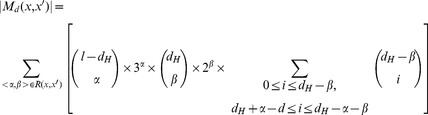
(3)


Moreover, as the process of verifying candidate motifs is to frequently calculate the Hamming distance between two *l*-mers, an efficient method is used in PairMotif to calculate *d_H_*(*x*, *x*′). First, convert *x* and *x*′ to integers *x_b_* and *x*′*_b_* by encoding each character of *x* and *x*′ to a 2-bit. Second, compute the bitwise exclusive disjunction of *x_b_* and *x*′*_b_*, denoted by *X*. Third, obtain *d_H_*(*x*, *x*′), which is the number of 2-bits that are not 00 in *X*, by searching a 256 byte table ⌈*l*/4⌉ times. At each position of the table, we cache the number of 2-bits that are not 00 in the associated 8-bit. In the implementation of PairMotif, all *l*-mers in *S* are represented as integers and cached for skipping the first step of this method. Therefore, in practice, this method is four times faster than comparing two *l*-mers directly. The details of this method and the evaluation of its speedup are included in the Text S2.

**Table 2:** Algorithm complexities.

Algorithm	Time complexity	Space complexity
PMSprune	*O*(*tn* ^2^4*^l^p_d_*)	*O*(*tn* ^2^)
iTriplet	*O*(*tn* ^3^ *l* ^2^ *d* ^3^ *p*_2*d*_^3^)	*O*(4*^l^p_d_*)
PMS5	*O*(*L*+*tn* ^3^ *d*4*^l^p_d_* ^3^)	*O*(*l* ^5^ *d* ^3^)
PairMotif	*O*(*tn* ^3^4*^l^p_d_* ^2^ *p*_2*d*_^2^)	*O*(*tn*)

This table shows the time and space complexities of PairMotif and that of other famous exact algorithms. Note that, *t* is the number of sequences; *n* is the sequence length; *p_k_* is the probability that the Hamming distance between two random *l*-mers is not more than *k*; *L* represents the time to load the ILP table of PMS5, which is about 50 seconds [22].

## Results and Discussion

We mainly compare the time performance of PairMotif with that of other famous exact algorithms, since all exact algorithms report the same results with different time overheads.

**Table 3:** Time comparison on fixed 2*d* neighborhood probability.

Algorithm	(12, 3)	(15, 4)	(18, 5)	(21, 6)	(24, 7)	(27, 8)	(30, 9)
RISOTTO	25 s	3.8 m	30.3 m	4.1 h	-o	-o	-o
PMS5	17 s	28 s	2.4 m	2.5 m	2.4 m	-e	-e
iTriplet	2.9 m	3.1 m	3.8 m	4.2 m	4.9 m	5.9 m	7.4 m
PMSprune	1 s	2 s	6 s	11 s	19 s	35 s	50 s
PairMotif	2 s	2 s	3 s	5 s	11 s	24 s	47 s

Time units, s: seconds; m: minutes; h: hours. Note, -o: over 10 hours; -e: memory error.

### Time and Space Analysis

Let *p_k_* denote the probability that the Hamming distance between two random *l*-mers is not more than *k* (0< *k* < *l*):
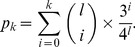
(4)


The time complexity of PairMotif is analyzed by estimating the number of candidate motifs and the number of *l*-mers to be scanned in verifying each candidate motif. PairMotif loops through *O*(*n* ^2^) pairs of *l*-mers. For each pair of *l*-mers *x* and *x*′, the probability that a random *l*-mer *y* becomes a candidate motif is Prob.[*d_H_*(*y*, *x*) ≤ *d* & *d_H_*(*y*, *x*′) ≤ *d*] = 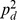, so the size of *M_d_*(*x*, *x*′) is approximately equal to 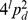. Thus, the total number of candidate motifs is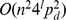. Furthermore, there are *O*(*tn*) potential *l*-mers to be scanned in verifying each candidate motif. After being filtered by both Rule 1 and Rule 2, each remaining *l*-mer *z* satisfies at least the condition that *d_H_*(*z*, *x*) ≤2*d* and *d_H_*(*z*, *x*′) ≤2*d*. Hence, the number of *l*-mers to be scanned is 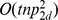. Based on the above considerations, the time complexity of PairMotif is 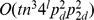.

**Table 4:** Time comparison on different 2*d* neighborhood probability.

(*l*, *d*)	Neighborhood Probability	RISOTTO	PMS5	iTriplet	PMSprune	PairMotif
(29, 8)	0.016	-o	-e	21 s	1 s	1 s
(9, 2)	0.049	3 s	14 s	2.6 m	1 s	1 s
(23, 7)	0.078	-o	2.6 m	19.3 m	2.3 m	2.2 m
(28, 9)	0.138	-o	-e	3.6 h	1.1 h	2.9 h
(19, 6)	0.175	7.5 h	3.0 m	1.9 h	5.9 m	4.0 m
(27, 9)	0.213	-o	-e	-o	-o	7.9 h
(18, 6)	0.283	-o	7.1 m	-o	29.6 m	12.1 m
(15, 5)	0.319	1.3 h	4.1 m	-o	8.7 m	4.7 m
(17, 6)	0.426	-o	31.3 m	-o	1.8 h	53.3 m
(19, 7)	0.534	-o	1.4 h	-o	-o	8.6 h

Time units, s: seconds; m: minutes; h: hours. Note, -o: over 10 hours; -e: memory error.

PairMotif requires little storage for implementation. In PairMotif, all *l*-mers in *s*_1_ are traversed with each of them processed independently. After processing one *l*-mer, the associated memory can be released, and we just need to consider the memory requirement for processing one *l*-mer *x*. Specifically, we store the (*t−*1)(*n-l+1*) *l*-mers in *s*_2_, …, *s_t_* and the Hamming distances from *x* to these *l*-mers. Therefore, the space complexity of PairMotif is *O*(*tn*).

**Figure 4:**
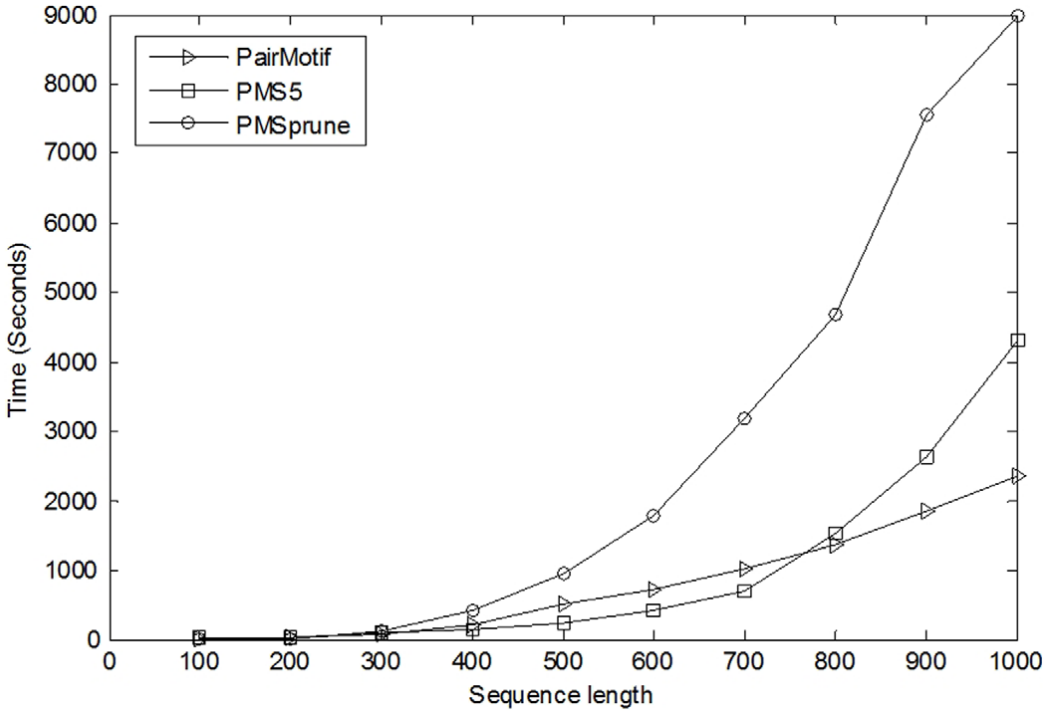
Time comparison on different sequence lengths. This figure compares PairMotif with two famous algorithms PMS5 [22] and PMSprune [20] on different sequence lengths on the instance (18, 6). The x-axis shows the sequence lengths. The y-axis shows the running times.

Table 2 shows the time and space complexities of PairMotif and those of several famous exact algorithms, such as PMSprune [20], iTriplet [21] and PMS5 [22]. PairMotif requires the least amount of storage space. Particularly, the space complexity of PairMotif and that of PMSprune, which depend on *t* and *n*, are fixed on different PMS instances; whereas the storage requirement of iTriplet and that of PMS5, which depend on *l* and *d*, may be unrealistic on the PMS instances with large values of *l* and *d*. For time complexity, PairMotif outperforms PMSprune because the ratio of their time complexities, 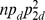, is less than 1. It also outperforms iTriplet on most PMS instances except for those with very large values of *l*. Moreover, PairMotif shows its performance advantage over PMS5 when *l* is small (*l* <15); in this case, the time overhead of loading the ILP table becomes the limiting factor in the performance of PMS5.

**Table 5:** Experimental results on real biological data.

Data set	(*l*, *d*) used	Amount of (*l*, *d*) motifs	Motif
preproinsulin	(15, 2)	10^2^	CAGCCTCAGCCCCCC^a^
			TG***CAGCCTCAGCCCC*** b
			GAAATTG***CAGCCTCA*** c
			TG***CAGCCTCAGCCCC*** d
DHFR	(11, 2)	10^3^	ATTTCGCGCCA^a^
			CATCGTCGCCG^b^
			***GCGCCA***AACTT^c^
			***TCGCGCCA***AAC^d^
c-fos	(9, 2)	10^4^	CCANATTNG^a^
			GCCTCCCCC^b^
			C***CTATTTGG***A^ce^
			GTTGGCTGC^d^
metallothionein	(15, 2)	10^1^	CTCTGCACRCCGCCC^a^
			***TCTGCACCCGGCCC***C^b^
			***CTCTGCACCCGGCAC*** c
			***TCTGCACCCGGCCC***C^d^
Yeast ECB	(16, 3)	10^1^	TTTCCCNNTNAGGAAA^a^
			***TTACCCATTTAGGAAA*** b
			***TTACCCATTAAGGAAA*** c
			***TTTCCCTTTTAGGAAA*** d

a The published motif.

b The predicted motif obtained by using consensus score.

c The predicted motif obtained by using relative entropy.

d The predicted motif obtained by using sequence specificity.

e The corresponding (*l*, *d*) used is (10, 2).

### Test on Simulated Data

We adopt the simulated data sets used in [6]. Generate a motif of length *l* and *t* identically distributed sequences of length *n*. Then, for each sequence *s*, implant a random motif instance, which differs from the motif in at most *d* positions, to a random position in *s*.

**Figure 5:**
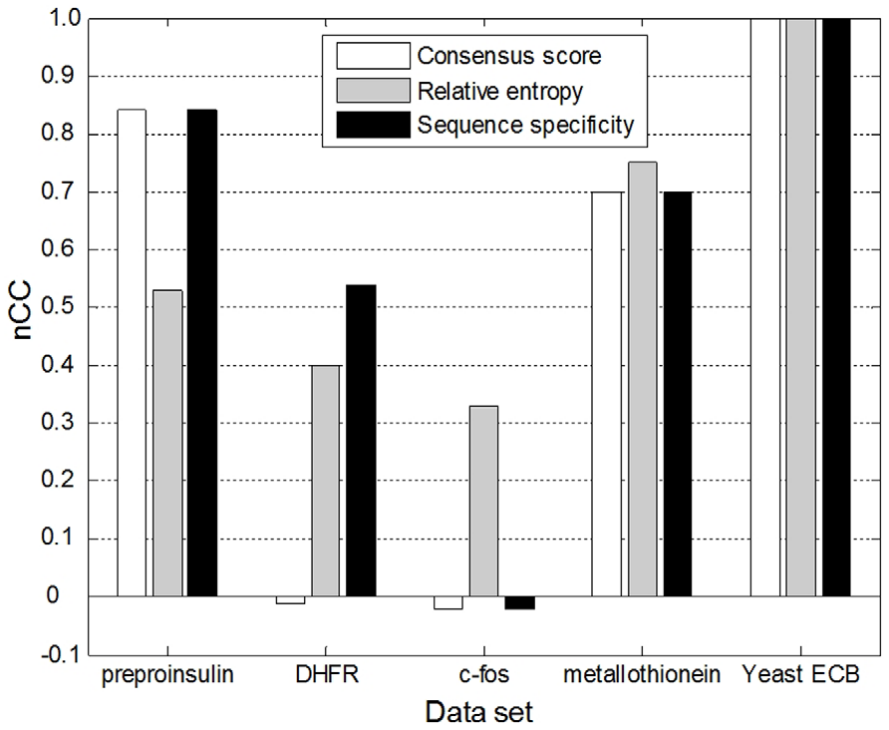
Comparison of predicted motifs under different objective functions. The x-axis shows the data sets used in our experiments. For each data set, we obtain three predicted motifs in terms of three objective functions. The y-axis shows the value of nucleotide-level correlation coefficient for each predicted motif.

In experiments, we fix *t* = 20 and *n* = 600, and compare the performance of PairMotif with that of several recently proposed exact algorithms, such as RISOTTO [18], PMSprune [20], iTriplet [21] and PMS5 [22], by varying *l* and *d* values (PMS instances). For the motif length *l*, we consider its value ranging from 9 to 30, as the binding sites are short DNA segments. To select a group of PMS instances to carry out comparisons, we use the 2*d* neighborhood probability *p*_2*d*_ calculated by (4) to indicate the weakness of a PMS instance. The larger the value of *p*_2*d*_, the weaker the corresponding PMS instance. All algorithms are performed in the same experimental environment with a 2.67 GHz processor and a 4 Gbyte memory. And the average running times are derived by executing different algorithms on ten simulated data sets.

At first, the comparisons are carried out on fixed 2*d* neighborhood probability *p*_2*d*_. Table 3 gives the running times of these algorithms on the PMS instances with the value of *p*_2*d*_ around 0.05, which is approximately the same as the *p*_2*d*_ value of the instance (15, 4). The results confirm with the time complexities in Table 2. PairMotif achieves the best execution time over other algorithms, and PMSprune is the second fastest. Although iTriplet exhibits stable running times, it does not show its performance advantage. PMS5 is defeated either because of the extra time overhead for loading ILP table or its high storage requirement [22]. RISOTTO is very sensitive to the value of *l*, and its running time increases exponentially when *l* increases.

Second, the running times of these algorithms are compared on a group of PMS instances with different probability *p*_2*d*_ that ranges from 0.01 to 0.5. We do not consider the probability *p*_2*d*_ whose value exceeds 0.5, as the corresponding motif is so degenerate that although exact algorithms can report all (*l*, *d*) motifs, it is difficult to distinguish the planted motif from spurious motifs. The running times are shown in Table 4. The performance of RISOTTO is the worst due to the strong sensitivity to the value of *l*. The iTriplet algorithm fails to solve the PMS instances with *p*_2*d*_ >0.2 because the value of *p*_2*d*_ severely affects its time complexity. PMSprune can solve most of these PMS instances in a short time except for the instances (27, 9) and (19, 7). PMS5 works well on the PMS instances with large *p*_2*d*_, whereas fails on the instances (29, 8), (28, 9) and (27, 9) because of memory limits. For PairMotif, all these PMS instances can be solved within 10 hours. Particularly, among these algorithms, only PairMotif can solve the instance (27, 9) while other algorithms fail because either a large amount of memory or execution time is required.

Moreover, it should be noticed that for a specific PMS instance, the longer the input sequences, the weaker the instance. It is therefore necessary to compare the performance of algorithms on different sequence length *n* given a PMS instance. To carry out comparisons, we select two algorithms PMS5 and PMSprune besides PairMotif, and then perform them on the well-known instance (18, 6) by varying *n* from 100 to 1000. Figure 4 plots the running times of these algorithms against the increase of *n*. The running time of PairMotif is almost linearly related to the sequence length; whereas the running time of PMS5 and that of PMSprune increase sharply as the sequence length increases, especially for PMSprune. The reason why the performance of PairMotif is stable over the sequence length is that PairMotif has strong ability of filtering scanned *l*-mers. That is, the remaining *l*-mers to be scanned after filtering are so few that their volume is almost unchanged for different sequence lengths. Neither PMS5 nor PMSprune possesses this property. Thus, although PairMotif does not outperform PMS5 when the input sequences are short, it does so when *n* >700.

### Test on Biological Data

To test the validity of PairMotif, we identify the known transcription factor binding sites in five real data sets discussed in [28], including preproinsulin, DHFR, c-fos, metallothionein and Yeast ECB (these data sets are included in the Dataset S1). These data differ substantially from the simulated data.

PairMotif reports all (*l*, *d*) motifs. To obtain one predicted motif, a specific objective function is needed to rank the reported motifs, and the motif with maximum score is selected. In our experiments, three objective functions (consensus score [12], relative entropy [29] and sequence specificity [30]) suitable for exact algorithms are used to obtain three predicted motifs for each data set.

Table 5 shows the results of our experiments. The (*l*, *d*) used for each data set is selected as follows: the value of *l* is fixed as the length of the published motif; the value of *d* is the minimum needed to ensure that the reported (*l*, *d*) motifs will contain the real motif. The third column gives the order of magnitude of the (*l*, *d*) motifs reported by PairMotif. The last column shows the predicted motifs selected from the reported (*l*, *d*) motifs by using three objective functions. The italicized part of each predicted motif indicates the part overlapped with the published motif.

PairMotif ensures that each predicted motif is optimal under the associated objective function. On this basis, the prediction accuracy depends on the used objective function. To evaluate each predicted motif, the nucleotide-level correlation coefficient (*nCC*) [31] is adopted:
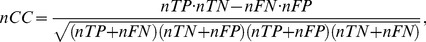
(5)where *nTP*, *nTN*, *nFN* and *nFP* are the number of true/false positive/negative predicted nucleotides. The use of correlation coefficient allows an integrated assessment of sensitivity and specificity.

Figure 5 compares the predicted motifs under different objective functions by showing their *nCC*s. The value of *nCC* ranges from −1 (indicating perfect anticorrelation) to +1 (indicating perfect correlation); the larger the value of *nCC*, the higher the accuracy of the predicted motif. For the five real data sets, the result accuracy under relative entropy and sequence specificity is obviously higher than that under consensus score. Nevertheless, no single objective function outperforms the others for every data set. For example, sequence specificity is well used for preproinsulin and DHFR, but leads to lower accuracy compared with relative entropy for c-fos and metallothionein. From the above analysis, PairMotif provides a good foundation for obtaining the real motif under a given objective function. If the objective function is able to assign the maximum score to the real motif, the real motif will be quickly found by PairMotif.

### Conclusions

DNA motif search is a challenging problem in computer science and bioinformatics. In this paper, we propose a new combinatorial algorithm, PairMotif, which restricts the search space of motif search by generating candidate motifs from multiple pairs of *l*-mers. PairMotif requires a small space complexity of *O*(*tn*), where *t* is the number of input sequences and *n* is the sequence length. It has a stable time performance over the sequence length, and it can solve most PMS instances in a reasonably short amount of time. Experimental results on real biological data sets show that PairMotif provides a good foundation for obtaining the real motif under a given objective function.

In PairMotif, all *l*-mers in *s*_1_ are traversed and each *l*-mer is processed independently. Therefore, PairMotif has a good feature of parallel computing, which allows it to use the strengths of parallel and distributed systems to improve the efficiency and quality of motif finding. Moreover, since all candidate motifs are traversed one by one in the current version of PairMotif, the performance of PairMotif can still be improved by using branch and bound in the process of traversing candidate motifs.

## Supporting Information

Text S1 **The correctness of filtering rule 2.**
(DOC)

Text S2 **Method for calculating Hamming distance between two** ***l*** **-mers.**
(DOC)

Dataset S1 **The real biological data sets used in our experiments, including preproinsulin, DHFR, c-fos, metallothionein and Yeast ECB, which are discussed in** [**28**] **.**
(RAR)

Program S1 **The executable program of algorithm PairMotif.**
(RAR)
