# Supplementary material for: PairMotif: A New Pattern-Driven Algorithm for Planted (l, d) DNA Motif Search
Source: PLoS One. 2012 Oct 31;7(10):e48442. doi: 10.1371/journal.pone.0048442 (PMC3485246; doi:10.1371/journal.pone.0048442)
Supplement: Text S1 — The correctness of filtering rule 2. (DOC) [file pone.0048442.s001.doc]

### Correctness of Filtering Rule 2

Let us prove the correctness of Rule 2. For a 2-tuple <*α*, *β*> ∈ *R*(*x*, *x*’), let *M_d_*<*_α_*, *_β_*>(*x*, *x*’) denote the subset of *M_d_*(*x*, *x*’) that contains all such candidate motifs *y* ∈ *M_d_*(*x*, *x*’) with *R*(*x*, *x*’, *y*) = <*α*, *β*>. Rule 2 implies that, *z* is not an instance of any motif in *M_d_*<*_α_*, *_β_*>(*x*, *x*’) if *abs*(|*P*_100_(*x*, *x*’, *z*)| - *α*) + *abs*(|*P*_000_(*x*, *x*’, *z*)| - *β*) > *d.* We only need to prove that *abs*(|*P*_10_(*x*, *x*’, *z*)| - *α*) + *abs*(|*P*_00_(*x*, *x*’, *z*)| - *β*) represents the minimum of the distance from *z* to all candidate motifs in *M_d_*<*_α_*, *_β_*>(*x*, *x*’) with the error value <*α*, *β*>. This is because if the minimum is larger than *d*, then *z* would not be an instance of any motif in *M_d_*<*_α_*, *_β_*>(*x*, *x*’).

At first, let us discuss the minimum with the error value *α* in the positions of *P*_1_(*x*, *x*’) by considering the following three cases:

(1) |*P*_10_(*x*, *x*’, *z*)| - *α* > 0. The minimum will occur when *P*_10_(*x*, *x*’, *z*) contains all *α* positions selected in step 1 of traversing candidate motifs, and the minimum is |*P*_10_(*x*, *x*’, *z*)| - *α* = *abs*(|*P*_10_(*x*, *x*’, *z*)| - *α*).

(2) |*P*_10_(*x*, *x*’, *z*)| - *α* = 0. The minimum will occur when the positions in *P*_10_(*x*, *x*’, *z*) are exactly the *α* positions selected in step 1 of traversing candidate motifs, and the minimum is 0 = *abs*(|*P*_10_(*x*, *x*’, *z*)| - *α*).

(3) |*P*_10_(*x*, *x*’, *z*)| - *α* < 0. The minimum will occur when the positions in *P*_10_(*x*, *x*’, *z*) are contained in the *α* positions selected in step 1 of traversing candidate motifs, and the minimum is *α - |P*_10_(*x*, *x*’, *z*)|= *abs*(|*P*_10_(*x*, *x*’, *z*)| - *α*).

Taking these cases into account, the minimum of the distance from *z* to all candidate motifs in *M_d_*<*_α_*, *_β_*>(*x*, *x*’) with the error value *α* is *abs*(|*P*_10_(*x*, *x*’, *z*)| - *α*) in the positions of *P*_1_(*x*, *x*’).
