# Supplementary material for: PairMotif: A New Pattern-Driven Algorithm for Planted (l, d) DNA Motif Search
Source: PLoS One. 2012 Oct 31;7(10):e48442. doi: 10.1371/journal.pone.0048442 (PMC3485246; doi:10.1371/journal.pone.0048442)
Supplement: Text S2 — Method for calculating Hamming distance between two l -mers. (DOC) [file pone.0048442.s002.doc]

### Method for calculating Hamming distance between two l-mers

Given two *l*-mers *x* and *x*', our method goes through the following three steps. First, convert *x* and *x*' to integers *x_b_* and *x*'*_b_* by encoding each character of *x* and *x*' to a 2-bit (two consecutive bits); we deal with only four characters, namely A, G, C and T, and 2 bits are enough to encode them. Second, compute the bitwise exclusive disjunction (XOR) of *x_b_* and *x*'*_b_*, denoted by *X*. Third, sum values returned by searching table ⌈*l*/4⌉ times. The Hamming distance between *x* and *x*' equals the number of 2-bits that are not 00 in *X*. We use an array as a lookup table, denoted by *T*[0] … *T*[255], in which each position corresponds to a unique 8-bit and caches the number of 2-bits that are not 00 in the associated 8-bit. *X* is traversed from right to left by visiting 8-bit each time. For every 8-bit, we take its corresponding decimal integer as the index to search lookup table, and add all returned values together to get the final distance. Note that, the memory overhead (256 bytes) of the lookup table is negligible.

The pseudocode for our method is presented as a procedure called CALCULATE-HAMMING-DISTANCE. Line 1 initializes the variables *d_H_*, which holds the Hamming distance obtained so far, *x_b_* and *x*'*_b_*, holding the corresponding integers of the two input *l*-mers. Lines 2 - 5, line 6 and lines 7 - 10 correspond to the first, second and third step of our method, respectively. The result is output in line 11. In the procedure, &, |, ^, << and >> represent bitwise AND, bitwise OR, bitwise XOR, left shift and right shift, respectively.

**CALCULATE-HAMMING-DISTANCE**

**Input:** two *l*-mers *x* and *x*', lookup table *T*[0..255]

**Output:** the Hamming distance between *x* and *x*'

1: *d_H_* ← 0, *x_b_* ← 0, *x*'*_b_* ← 0

2: **for** *i* ← 1 **to** *l* **do**

3: *num* ← corresponding 2-bit of *x*[*i*], *num*' ← corresponding 2-bit of *x*'[*i*]

4: *x_b_* ← *x_b_* << 2, *x*'*_b_* ← *x*'*_b_* << 2

5: *x_b_* ← *x_b_* | *num*, *x*'*_b_* ← *x*'*_b_* | *num*'

6: *X* ← *x_b_* ^ *x*'*_b_*

7: **for** *i* ← 1 **to** ⌈*l*/4⌉ **do**

8: *index* ← *X* & 255

9: *d_H_* ← *d_H_* + *T*[*index*]

10: *X* ← *X* >> 8

11: return *d_H_*

The time complexity of our method is *O*(*l*). The first step (converting *l*-mers to their binary equivalents) becomes the bottleneck, which has the same complexity of comparing two *l*-mers directly and makes our method work even worse.

However, this drawback can be overcome when our method is used in PairMotif. Specifically, the first step is skipped by using cache technique in the implementation of PairMotif. All *l*-mers in input sequences are converted to integers in advance and these integers are cached. Each candidate motif is also in an integer form, because the process of traversing candidate motifs is done by operating integers in the program design. Thus, when we calculate the Hamming distance between a candidate motif and a scanned *l*-mer, we obtain two integers directly and avoid the step for converting *l*-mers to their binary equivalents. Because of the cache technique, only *O*(*tn*) *l*-mers need to be converted to integers and this quantity is negligible compared to the total number of scanned *l*-mers in PairMotif.

Based on these considerations, when our method is used in PairMotif, its time complexity is determined by the third step. It is *O*(*l*/4). Therefore, in practice, our method is four times faster than comparing two *l*-mers directly.
